# Patient-reported health outcomes after total hip and knee surgery in a Dutch University Hospital Setting: results of twenty years clinical registry

**DOI:** 10.1186/s12891-017-1455-y

**Published:** 2017-03-03

**Authors:** Philip J. van der Wees, Joost J. G. Wammes, Reinier P. Akkermans, Jan Koetsenruijter, Gert P. Westert, Albert van Kampen, Gerjon Hannink, Maarten de Waal-Malefijt, B. Willem Schreurs

**Affiliations:** 1 Radboud University Medical Center, Radboud Institute for Health Sciences; Scientific Institute for Quality of Healthcare, P.O. Box 9101, 6500 HB Nijmegen, The Netherlands; 2 Radboud University Medical Center, Radboud Institute for Health Sciences; Primary and Community Care, P.O. Box 9101, 6500 HB Nijmegen, The Netherlands; 3 Department of Orthopedics, Radboud University Medical Center, P.O. Box 9101, 6500 HB Nijmegen, The Netherlands

**Keywords:** Outcomes measurement, Patient-reported outcomes, Total hip replacement, Total knee replacement

## Abstract

**Background:**

Patient-Reported Outcome (PRO) measurement is a method for measuring perceptions of patients on their health and quality of life. The aim of this paper is to present the results of PRO measurements in total hip and knee replacement as routinely collected during 20 years of surgery in a university hospital setting.

**Methods:**

Data of consecutive patients between 1993 and 2014 were collected. Health outcomes were measured pre-surgery and at 3, 6, and 12 months post-surgery. Outcomes for hip replacement were measured with the Harris Hip Score (HHS) and Oxford Hip Score (OHS). Outcomes for knee replacement were measured with the Western Ontario and McMaster Universities Arthritis Index (WOMAC) and the Knee Society Score (KSS). A Visual Analog Scale (VAS) for pain was used. Absolute and relative Minimal Clinically Important Differences (MCID) were estimated. Generalized estimating equation analysis was used for estimating mean outcomes. Trends over time were analyzed.

**Results:**

The database contained 2,089 patients with hip replacement, and 704 patients with knee replacement. Mean HHS and OHS scores in primary hip replacement at 12 months post-surgery were 86.7 (SD: 14.5) and 41.1 (SD: 7.5) respectively. Improvements on the HHS based on absolute MCID was lower for revisions compared to primary hip replacements, with 72.4% and 87.0% respectively. Mean WOMAC and KSS scores in knee replacement at 12 months post-surgery were 21.5 (SD: 18.2) and 67.0 (SD: 26.4) respectively. Improvements based on absolute MCID were lowest for the KSS (62.6%) and highest for VAS pain (85.6%). Trend analysis showed a difference in 1 out of 24 comparisons in hip replacement and in 2 out of 9 comparisons in knee replacement.

**Conclusions:**

The functional status of a large cohort of patients significantly improved after hip and knee replacement based on routine data collection. Our study shows the feasibility of the routine collection of PRO data in patients with total hip and knee replacement. The use of PRO data provides opportunities for continuous quality improvement.

## Background

Hip and knee osteoarthritis are leading causes of disability resulting in joint pain and stiffness [1, 2]. Joint replacement is a recommended intervention if disability is significant and conservative management is ineffective [3]. Prevalence of hip and knee joint replacement in the U.S. population is estimated at 2.5 and 4.7 million respectively [4]. Patient-reported outcomes (PRO) are important variables to quantify the results of surgical intervention after total hip and knee replacement [5, 6].

PRO measurement is a standardized method for measuring perceptions of patients on their health and health-related quality of life in relation to health care provided. Clinicians can use PROs to focus on a patient’s individual health goals and to guide diagnostic and treatment decisions. Aggregated across patients, PRO results can be used to guide efforts to improve clinical quality, for public reporting, and for value-based payments [7–10]. Large cohorts have been described in (inter)national registries for monitoring patients after total hip and knee replacement [11–13]. However, the use of PRO data in registries is still limited [14, 15]. A body of knowledge needs to be built to understand outcomes in non-controlled settings.

The department of Orthopedics at Radboudumc has established a clinical registry in the mid-90s to collect routine data of clinical and patient-reported health outcomes of patients after total hip and knee replacement. The aim of this paper is to present the results of PRO measurements as routinely collected during 20 years of surgery. The prolonged timeframe with routine data collection provides an excellent basis for building knowledge, and the main objective of the paper is to provide normative PRO data in real world settings.

## Methods

### Design, setting and participants

Radboudumc is one of the eight University Medical Centers in the Netherlands. The Orthopedic Department established a clinical registry in 1993 for the routine collection of health outcomes prior to and after total hip and knee replacement. Patients indicated for surgery were routinely referred to a clinical scoring station for measurements pre- and post-surgery follow up. The data was collected and stored in a local database at the hospital. This observational study presents data of consecutive patients that received total hip and knee replacement between October 1993 and February 2014.

### Patient-reported health outcomes

Health outcomes in total hip replacement were measured with the Harris Hip Score (HHS), the Oxford Hip Score (OHS), a visual analog scale (VAS) for pain in rest, and a VAS for pain during exercise. The HHS contains eight items for pain, function, walking aids, walking, stair walking, shoe lacing, sitting, and public transportation. The total score is 0 points if a patient has major problems on all items and 100 points if a patient has no problems at all [16]. The OHS contains 12 items related to pain, physical functioning and (social) activities [16]. We used the adapted scoring system of Murray where 48 points is the best score and 0 points is the worst score [17].

Health outcomes in total knee replacement were measured with the Western Ontario and McMaster Universities Arthritis Index (WOMAC), the Knee Society Score (KSS), and a visual analog scale (VAS) for pain. The WOMAC is a questionnaire containing 24 items in three domains: pain, joint stiffness, physical functioning. The total score is 96 points if a patient has major problems on all items [18, 19]. The KSS was developed to rate both the knee prosthesis function and patients’ functional abilities after total knee replacement. The functional abilities score is related to walking, walking stairs, and walking aids with a maximum score of 100 points if patients experience no problems in their functioning [20]. The KSS was revised in 2011 expanding the score to five components [21]. In our study we used the original scoring system for functional ability.

The VAS score is a continuous scale comprised of a line, 100 mm in length, anchored by two descriptors, one for each symptom extreme. A score of 0 represents “no pain” and a score of 100 represents “worst imaginable pain” [22].

### Measurements

At the Orthopedic Department of Radboudumc, measurements were routinely conducted at the clinical scoring station under supervision of a medical intern. Data were collected directly following the indication for surgery and during routine visits at 3, 6 and 12 months post-surgery. In addition, data on observed complications during and following surgery were collected.

### Data analysis

We used descriptive analysis to obtain insight in patient characteristics and complications. We used a well-defined classification system for determining complications frequently used in the Netherlands [23]. In this complication system both surgery related orthopedic complication (e.g. infection, luxation, fracture) are registered as well as other medical complications (e.g. cardiac, psychiatric). Complications were registered up to 1 year after surgery.

Measurements were categorized as follows: pre-surgery (between 6 months pre-surgery and date of surgery); 3-months (between 1.5 and 4.5 months post-surgery); 6 months (between 4.5 and 9 months post-surgery); and 12 months (between 9 and 15 months post-surgery).

Paired t-tests were used to compare outcomes preoperatively and after 12-months follow-up. In addition, we estimated minimal clinically important differences (MCID). The MCID is defined as the minimal change on a score that is important to the patient, and is used as parameter to enable clinical interpretation of change scores. We used two methods for calculating the proportion of patients who reached the threshold for a MCID. First, we assigned a dichotomous score for a clinically important improvement per outcome, based on an absolute MCID cut off point [24, 25]. Second, we calculated a dichotomous score per outcome based on 30% improvement from baseline [26–28] To avoid ceiling effects we only included patients with potential improvement based on the absolute and relative cut-off points. Minimally clinically important differences between baseline and follow-up scores were calculated at T = 6 months (scores at 6 months post-operative compared with pre-operative scores), and at T = 12 months (scores at 12 months post-operative compared with pre-operative scores).

We estimated MCID after total hip replacement based on HHS, OHS, and VAS outcomes. HHS scores have been categorized as follows: >90 excellent; 80–89 good; 79–79 fair, and <70 poor [16, 29]. We categorized OHS scores of > 41 as excellent, 34–41 good, 27–33 fair, and <27 poor [5, 17, 30, 31]. Based on consensus we used an improvement of at least one category as MCID for the HHS and OHS.

We estimated MCID after total knee replacement based on WOMAC, KSS, and VAS outcomes. The MCID for the WOMAC has been estimated at around 15–20 points [18], with relative improvements of 21–41% for its subscales [32–34]. We used a MCID of 20 points based on consensus in the project team. KSS scores have been categorized as excellent (>80 points), good (70–79 points), moderate (60–69 points) and poor (<60 points) [35, 36]. Based on consensus we used an improvement of at least one category as MCID for the KSS. For VAS pain a MCID of 20 mm was used [34].

We used generalized estimating equation (GEE) analysis for estimating the mean outcomes. A main asset of GEE analysis is that it uses all observations within one subject, thus reducing potential bias due to missing data [37]. GEE analysis is based on repeated measurement within subjects, allowing for modeling the within-subject residuals to correct for patient (gender, age) and surgical (complications) characteristics as confounding variables. We included baseline scores in the model by using all observations within one subject in the GEE analysis. We used registered complications during and post-surgery and dichotomized them for each patient: 0 complications versus ≥1 complication.

To analyze trends over time we used 5-year timeframes: 1993–1999; 2000–2004; 2005–2010; 2011–2014 - with 2011–2014 as reference - and included these as independent variables in the full GEE-models. This resulted in 24 comparisons for primary hip replacement and revisions; and nine comparisons for total knee replacement.

## Results

### Patient and surgical characteristics

Patient and surgical characteristics in total hip surgery are summarized in Table 1. This database contained 2,089 unique patients, with 778 men (37%) and 1311 women (63%). Total number of surgical total hip procedures was 2,545; with 1,877 primary replacements and 668 revisions. In 19.7% of all surgical procedures one or more complications was registered.

**Table 1:** Patient and surgical characteristics of total hip replacement

Patient characteristics	N (%)
Unique patients	2,089
Mean age (SD)	61.4 (15.6)
Age distribution	
0-30 years	98 (4.9%)
31-50 years	348 (17.5%)
51-75 years	1187 (59.6%)
≥ 76 years	359 (18.0%)
Sex: male/female	778 (37.2%)/1311 (62.8%)
Surgical characteristics	N (%)
Number of surgeries	2,545
Primary hip replacement	1877 (73.8%)
Revision	668 (26.2%)
Complications in primary hip replacement	339 (18.0%)^a^
Complications in revisions	163 (24.3%)^a^

^a^One or more complications in surgical procedures

Patient and surgical characteristics in total knee replacement are summarized in Table 2. This database contained 704 unique patients, with 250 men (35.5%) and 454 women (64.5%). The total number of primary total knee replacements was 799. The database did not contain any data of total knee revisions. In 13.4% of surgical procedures one or more complications was registered.

**Table 2:** Patient and surgical characteristics of total knee replacement

Patient characteristics	N (%)
Unique patients	704
Mean age (SD)	65.0 (12.0)
Age distribution	
0-30 years	9 (1.3%)
31-50 years	66 (9.7%)
51-75 years	465 (68.7%)
≥ 76 years	137 (20.2%)
Sex: male/female	250 (35.5%)/454 (64.5%)
Surgical characteristics	N (%)
Number of surgeries (primary knee replacement)	799
Complications	107 (13.4%)

### Health outcomes after total hip replacement

**Fig. 1:**
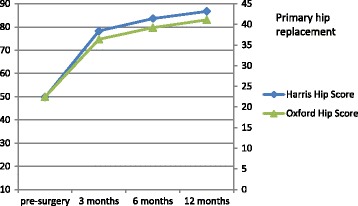
Uncorrected mean scores for HHS and OHS in primary hip replacement

**Fig. 2:**
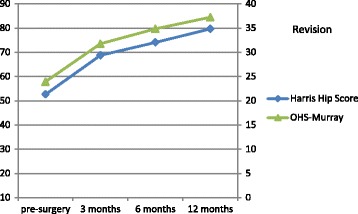
Uncorrected Mean scores for HHS and OHS in total hip revision

**Fig. 3:**
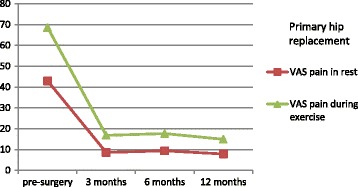
Uncorrected mean pain scores in rest and during exercise in primary hip replacement

**Fig. 4:**
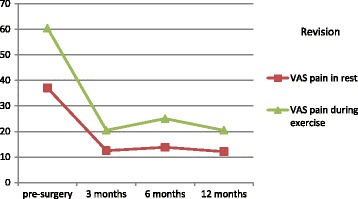
Uncorrected mean pain scores in rest and during exercise in total hip revision

Uncorrected scores for the HHS and OHS are presented in Table 3, and uncorrected scores for VAS pain are presented in Table 4. All patients had at least one measurement point at either pre-surgery or at one of the post-surgery follow-up measurements. The distribution of the number of 1, 2, and ≥3 measurement points was 30.5%, 27.4%, and 42.1% respectively. Mean HHS scores for primary hip replacement pre-surgery and at 12 months post-surgery were 49.7 (SD: 16.0) and 86.7 (SD: 14.5) respectively. For revisions the pre-surgery and 12-months post-surgery mean HHS scores were 52.6 (SD: 19.3) and 79.7 (SD: 17.1) respectively. Uncorrected mean scores at the different measurements for the HHS and OHS after primary hip replacement and revision are presented in Fig. 1 and 2 respectively. Uncorrected mean scores at the different measurements for pain before and after primary hip replacement and revision are presented in Fig. 3 and 4 respectively. The mean differences between baseline and 12 months follow-up were statistically significant for all outcomes (*p* < 0.001).

**Table 3:** Uncorrected scores and response rates of the Harris Hip Score and Oxford Hip Score

	Harris hip score						Oxford hip score					
	Primary			Revision			Primary			Revision		
	Score (SD)	N	%	Score (SD)	N	%	Score (SD)	N	%	Score (SD)	N	%
Pre-surgery	49.7 (16.0)	1252	67	52.6 (19.3)	268	40	22.4 (8.4)	780	41	23.9 (9.9)	196	29
3 months	78.3 (14.5)	794	42	68.6 (15.3)	360	52	36.4 (7.7)	797	42	31.7 (9.4)	382	56
6 months	83.6 (15.3)	699	37	74.0 (17.9)	313	47	39.2 (8.1)	681	36	34.8 (9.7)	314	47
12 months	86.7 (14.5)	789	42	79.7 (17.1)	314	47	41.1 (7.8)	781	42	37.2 (9.1)	315	47
Av. Response^a^			47			47			40			45

Primary: primary hip replacement; Response rates for primary hip replacement are based on *n* = 1877 surgical procedures; Response rates for revisions are based on *n* = 688 surgical procedures

^a^Average response rates per measurement point. All patients had at least one measurement point at either pre-surgery or at one of the post-surgery follow up measurements. The distribution of the number of 1, 2, and ≥3 measurement points was 30.5%, 27.4%, and 42.1% respectively

**Table 4:** Uncorrected scores of the VAS pain in total hip replacement^a^

	VAS pain in rest						VAS pain during exercise					
	Primary			Revision			Primary			Revision		
	Score (SD)	N	%	Score (SD)	N	%	Score (SD)	N	%	Score (SD)	N	%
Pre-surgery	43.0 (28.2)	806	43	37.0 (29.2)	204	30	68.6 (22.7)	803	43	60.4 (28.1)	202	30
3 months	8.6 (16.6)	842	45	12.6 (20.5)	397	58	16.8 (22.3)	842	45	20.5 (24.9)	397	58
6 months	9.4 (17.3)	716	38	13.8 (20.9)	330	49	17.7 (24.3)	715	38	25.0 (26.9)	330	49
12 months	7.9 (16.8)	805	43	12.1 (21.4)	324	48	14.9 (23.3)	804	43	20.4 (26.7)	324	48
Av Response^a^			42			46			42			46

*VSS* Visual Analog Scale; Primary: primary hip replacement; Response rates for primary hip replacement are based on *n* = 1877 surgical procedures; Response rates for revisions are based on *n* = 688 surgical procedures

^a^ Average response rates per measurement point. All patients had at least one measurement point at either pre-surgery or at one of the post-surgery follow-up measurements. The distribution of the number of 1, 2, and ≥3 measurement points was 30.5%, 27.4%, and 42.1% respectively

Table 5 shows the MCID based on absolute cut-off points. Improvements at 12 months after primary hip replacement were lowest for the HHS (87.0%) and highest for the OHS (93.1%). Improvements on the HHS at 12 months post-surgery based on MCID were substantially lower for revisions compared to primary hip replacement, with 72.4% and 87.0% respectively. Table 6 shows the MCID based on minimal 30% improvement in PROM-scores. The two methods for estimating MCID showed comparable improvements.

**Table 5:** Absolute MCID after total hip replacement

	Primary hip replacement^d^		Revisions^e^	
	T = 6 months	T = 12 months	T = 6 months	T = 12 months
Harris Hip Score^a^	84.1% (N = 371)	87.0% (N = 416)	62.2% (N = 90)	72.4% (N = 98)
Oxford Hip Score^b^	90.8% (N = 358)	93.1% (N = 378)	69.9% (N = 93)	77.2% (N = 92)
VAS pain (rest)^c^	89.1% (N = 311)	90.6% (N = 319)	72.7% (N = 77)	79.2% (N = 72)
VAS pain (exercise)^c^	87.8% (N = 368)	92.6% (N = 392)	70.2% (N = 94)	75.8% (N = 95)

*MCID* Minimal Clinically Important Differences, *HHS* Harris Hip Score, *OHS* Oxford Hip Score, *VAS* Visual Analog Scale

^a^HHS scores are categorized as >90 excellent; 80-89 good; 79-79 fair and <70 bad [16, 29]. We defined a clinically relevant improvement as one category improvement

^b^OHS scores are categorized as > 41 excellent, 34 -41 good, 27-33 fair, <27 bad [30, 31]. We defined a clinically relevant improvement as one category improvement

^c^We used a difference of 20 mm or more as clinically relevant improvement [34]

^d^Primary hip: HHS: 0.7% excluded because of ceiling. OHS: 1.5% excluded because of ceiling. VAS pain rest: 37.2% were excluded because of ceiling. VAS pain exercise: 4.5% were excluded because of ceiling

^e^Revision hip: HHS: 4.9% excluded because of ceiling. OHS: 5.6% excluded because of ceiling. VAS pain rest: 28.2% were excluded because of ceiling. 10.9% were excluded because of ceiling

**Table 6:** Relative MCID of 30% improvement after total hip replacement

	Primary hip replacement^b^		Revisions^c^	
	T = 6 months	T = 12 months	T = 6 months	T = 12 months
Harris Hip Score	84.5% (N = 373)	86.6% (N = 419)	59.3% (N = 91)	66.3% (N = 104)
Oxford Hip Score^a^	88.8% (N = 347)	91.5% (N = 365)	61.8% (N = 89)	67.1% (N = 85)
VAS pain (rest)^a^	88.9% (N = 348)	91.3% (N = 357)	74.4% (N = 73)	76.8% (N = 82)
VAS pain (exercise)^a^	84.5% (N = 375)	91.1% (N = 403)	67.4% (N = 95)	74.7% (N = 99)

*HHS* Harris Hip Score, *OHS* Oxford Hip Score, *VAS* Visual Analog Scale

^a^Patients with pre-operative score of 0 points were equaled to 1 point, because it is not possible to establish relative differences from a baseline value 0

^b^Primary hip replacement: HHS: 4.9% excluded because of ceiling. OHS: 4.7% excluded because of ceiling. VAS pain rest: 15.4% were excluded because of ceiling. VAS pain exercise: 2.2% were excluded because of ceiling

^c^Revisions: HHS: 12.7% excluded because of ceiling. OHS: 9.7% excluded because of ceiling. VAS pain rest: 24% were excluded because of ceiling. VAS pain exercise: 8.9% were excluded because of ceiling

### Health outcomes after total knee replacement

**Fig. 5:**
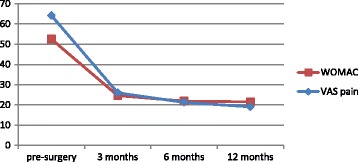
Uncorrected mean scores for the WOMAC and VAS pain in total knee replacement

Uncorrected scores for the WOMAC, KSS function score and VAS pain are presented in Table 7. All patients had at least one measurement point at either pre-surgery or at one of the post-surgery follow-up measurements. The distribution of the number of 1, 2 and ≥3 measurement points was 19%, 30.5%, and 50.5% respectively. Mean WOMAC scores for primary knee replacement pre-surgery and at 12 months post-surgery were 52.5 (SD: 16.3) and 21.5 (SD: 18.2) respectively. Mean scores on the KSS function score were 42.0 (SD: 22.1) pre-surgery and 67.0 (SD: 26.4) 12-months post-surgery. Uncorrected mean scores at the different measurements for the WOMAC and VAS pain before and after knee replacement are presented in Fig. 5. The mean differences between baseline and 12 months follow-up were statistically significant for all outcomes (*p* < 0.001).

**Table 7:** Uncorrected scores of WOMAC, KSS and VAS pain in total knee replacement^b^

	WOMAC			KSS function score			VAS pain		
		Response			Response			Response	
	Score (SD)	N	%^a^	Score (SD)	N	%^a^	Score (SD)	N	%^a^
Preoperative	52.5 (16.3)	385	46	42.0 (22.1)	435	54	64.2 (21.9)	409	51
3 months	24.6 (16.2)	454	57	57.2 (26.3)	486	60	26.0 (23.5)	485	61
6 months	21.9 (16.6)	395	49	64.7 (27.0)	424	53	21.3 (23.8)	422	53
12 months	21.5 (18.2)	450	56	67.0 (26.4)	479	60	19.1 (23.4)	473	59
Av. Response^b^			52			57			56

^a^Based on *n* = 799 surgical procedures

^b^Average response rates per measurement point. All patients had at least one measurement point at either pre-surgery or at one of the post-surgery follow-up measurements. The distribution of the number of 1, 2 and ≥3 measurement points was 19%, 30.5%, and 50.5% respectively

Table 8 shows the MCID based on absolute cut-off points. Improvements at 12 months were lowest for the KSS function score (62.6%) and highest for the VAS pain (85.6%). Table 9 shows the MCID based on minimally 30% improvement in PROM-scores. The two methods for estimating MCID showed comparable improvements.

**Table 8:** Absolute MCID after total knee replacement^d^

	T = 6 months	T = 12 months
WOMAC^a^	75.8% (*N* = 178)	80.2% (*N* = 177)
KSS function score^b^	58.7% (*N* = 213)	62.6% (*N* = 219)
VAS pain^c^	83.3% (*N* = 204)	85.6% (*N* = 201)

^a^Several criteria exist for estimating clinically important improvements in using the WOMAC. We used a pragmatically chosen cut-off point of at least 20 points improvement

^b^KSS scores have been categorized in >80 excellent; 70–79 good; 60–69 fair and <60 bad [36]. We defined a clinically relevant improvement as one category improvement

^c^We used a difference of 20 mm or more as clinically relevant improvement [34]

^d^WOMAC: 3.1% were excluded because of ceiling. KSS function score: 4.6% were excluded because of ceiling. VAS pain: 5.4% were excluded because of ceiling

**Table 9:** Relative MCID of 30% improvement after total knee replacement^b^

	T = 6 months	T = 12 months
WOMAC	82.5% (*N* = 200)	79% (*N* = 210)
KSS function score^a^	68.1% (*N* = 213)	68.5% (*N* = 219)
VAS pain^a^	80.7% (*N* = 212)	84.9% (*N* = 205)

^a^Patients with pre-operative score of 0 points were equaled to 1 point, because it is not possible to establish relative differences from a baseline value 0

^b^WOMAC: 0% were removed because of ceiling. KSS function: 4.6% were removed because of ceiling. VAS pain 2.9% were removed because of ceiling

### Determinants of health outcomes

In total hip replacement, comparisons in the GEE model showed better health outcomes for male patients with higher scores on the OHS and HHS; and less pain in rest and during exercise. Health outcomes were worse in patients with complications. The effects are presented in Table 10. The corrected scores showed a maximum difference of 2.0 points compared to the uncorrected scores, and the distribution of scores did not change between the different measurements.

**Table 10:** GEE estimates for gender and complications in total hip replacement

	Gender (male)		Complication (no)	
	Primary score (95%CI)	Revision score (95%CI)	Primary score (95%CI)	Revision score (95%CI)
HHS	4.6 (3.4,5.8) *p* < 0001	6.8 (4.4, 9.3) *p* < 0.001	3.6 (2.0, 5.2) *p* < 0.001	2.8 (0.005, 5.5) *p* = 0.05
OHS	3.3 (2.6, 4.) *p* < 0.001	4.4 (3.0,5.8) *p* < 0.001	1.8 (0.8, 2.8) *p* < 0.001	1.9 (0.3, 3.5) *p* = 0.02
VAS pain in rest	-3.6 (-5.3, -1.9) *p* < 0.001	-5.3 (-8.4, -2.2) *p* < 0.001	ns	ns
VAS pain during exercise	-3.7 (-5.7, -1.8) *p* < 0.001	-8.4 (-12.1,-4.7) *P* < 0.001	-3.6 (-6.2, -1.0) *p* = 0.008	ns

*GEE* Generalized Estimation Equation analysis, *HHS* Harris Hip Score, *OHS* Oxford Hip Score, *VAS* Visual Analog Scale; Primary: primary hip replacement; ns: not statistically significant

In total knee replacement, comparisons in the GEE model showed better scores for male patients on all outcomes. Age was only statistically significant in estimating outcomes of the KSS function score. All comparisons showed a worse score on all outcomes in patients with complications. The effects are presented in Table 11. The corrected scores showed a maximum difference of 3.5 points compared to the uncorrected scores, and the distribution of scores did not change between the different measurements.

**Table 11:** GEE estimates for gender, complications and age in total knee replacement

	Gender (male)	Complication (no)	Age
	Score (95% CI)	Score (95%CI)	Score (95%CI)
WOMAC	-5.4 (-7.5, -3.2) *p* < 0.001	-5.0 (-8.0, -1.9) *p* = 0.001	ns
KSS function score	11.4 (8.3-14.6) *p* < 0.001	10.9 (6.5-15.3) *p* < 0.001	-0.2 (-0.37,-0.11) *p* < 0.001
VAS pain	-3.6 (-6.5, -0.8) *p* = 0.01	-4.8 (-8.8, -0.8) *p* = 0.02	ns

*ns* not statistically significant

### Trends over time

The trend analysis over time after total hip replacement showed that VAS pain during exercise after primary hip replacement was significantly lower in the period 2000–2004, compared to the reference period 2011–2014. The trend analysis over time after total knee replacement showed that VAS pain was significantly lower in two comparisons for the periods 1996–1999 and 2000–2004, compared to the reference period.

## Discussion

Our study showed that the functional status of a large cohort of patients significantly improved after total hip and knee replacement, based on routine data collection in clinical practice. Male patients and patients without complications improved more than female patients and patients with complications. The two methods for MCID showed similar results. Trend analysis over time showed that patients had more pain after primary hip and knee replacement in earlier time periods compared to the reference period 2011–2014.

In total hip replacement the average HHS scores in our study at 12 months post-surgery are considered good [16, 29]. In a cohort of almost 600 patients similar HHS scores were found after primary hip replacement at 12 months post-surgery [38]. The average score on the OHS in primary total hip replacement at 12 months post-surgery is considered excellent [17], and comparable to outcomes of a cohort of almost 800 patients after primary hip replacement using the OHS [6].

Improvements in patient-reported outcomes after total knee replacement have been identified in several studies. A Canadian study included 298 patients for PRO measurement after total knee replacement [39]. Their data showed that patients significantly improved on the OKS and the KSS. A Swiss group of researchers analyzed data of 98 patients that were followed-up with PRO measurements after total knee replacement [40]. Their data showed lower pre-operative scores on the WOMAC and at 12 months follow-up than in our study.

We specified improvements by estimating clinically relevant improvements based on MCID. Our results show considerable variations in improvements in total hip and knee replacement based on mean scores on the outcome measures, while improvements were consistent over the two different methods for estimating MCID. This suggests that presenting MCID might be a good approach for presenting differences in outcomes within and between health care organizations.

Beswick et al found that at least 9% of patients with hip replacement and about 20% of patients with knee replacement report unfavorable long term pain outcome [41]. We did not quantify the share of patients with pain postoperatively. However, our findings are very much in line with Beswick’s, as we found that 91% of patients with primary hip replacement and 81% of patients with knee replacement reduced their pain scores by at least 30%. A significant share of patients thus experience pain after surgery, and improvements in the procedure and improved identification of patients eligible for surgery may be worthwhile.

Female gender and the incidence of complications were identified as determinants for lower functional outcomes. The difference between males and females has been identified before [38]. The reason for the better functioning of males after joint replacement is not clear but is assumed to be related to differences in health perceptions [42].

The GEE model showed only small differences between uncorrected and corrected data, without changes in the distribution between variables for the different measurements. This implies that missing data were randomly distributed across our cohort. Xie and colleagues also used a GEE model in estimating change scores and concluded that the magnitude of change scores on the selected health outcomes was similar to those with and without the adjustment of covariates [39].

The GEE modeling including different time frames showed no improvements in outcomes over time. In fact, two earlier time frames showed lower pain scores compared to the 2011–2014 reference period. Therefore we reject our hypothesis that outcomes after total knee replacement increased over time. We have no clear explanation for this. A study by Singh showed that functional limitations and pain worsened over time after primary knee replacement, also in contrast with their hypothesis [43]. A possible explanation may be that early discharge of patients has become more common over time, with a negative impact on patient functioning. During the whole period, we used cemented prosthesis in hip and knee replacement, without major changes in the surgical procedure.

The routine collection and presentation of PRO data after total hip and knee replacement serves several purposes. Clinicians and patients can use individual patient data to monitor progress over time. At the group level health outcomes can be used for quality improvement purposes and for presenting the results to the public. The department of orthopedics has decided to publish its data on their website to provide transparency to patients and stakeholders [44, 45]. The next step is to use the data for quality improvement purposes, e.g. via peer assessment of colleagues working in the same surgical team. The data can also be used for comparing outcomes between hospitals, although requirements for validity and reliability are high when comparing outcomes for accountability and appropriate case-mix adjustment is needed [46, 47].

A considerable amount of work is required making routine PRO measurement a success [48]. Our data show the feasibility of routine collection of PRO data in a hospital setting, and the data will be used for the Dutch national registry in joint replacement [49]. To our knowledge, this is the first study presenting PROs in thousands of orthopedic patients over a prolonged time frame. Therefore, it represents excellent reference material for assessing outcomes after surgery elsewhere.

Our study has several limitations. First, we estimated that the continuous data collection resulted in the inclusion of about half of all enrolled patients during our 20-year time frame. Second, the overall response rate of included patients was 50%; showing a large gap in data collection. Third, secular trends over time may have influenced our results. However, we found no major impact of trends over time. Our data showed a high percentage of complications, which may be explained by the broad definition of a complication we used; any unexpected medical event was reported including e.g. urinary infections.

PRO measurement could be an important addition to (inter)national registries by quantifying optimal outcomes after total hip and knee replacement procedures [15]. Our study shows the feasibility of the routine collection of PRO data in total hip and knee replacement. The data provides opportunities for continuous quality improvement, and for providing transparency of care in comparing outcomes between hospitals. An important aspect in managing the routine collection of data is ensuring high response rates. Future research should aim at interpreting outcomes for further improvements in the care of patients with hip and knee osteoarthritis.

## Conclusion

The functional status of a large cohort of patients significantly improved after hip and knee replacement based on routine data collection. This is the first study presenting PROs in thousands of orthopedic patients over a prolonged time frame. Therefore, it represents excellent reference material for assessing outcomes after surgery elsewhere. Our study shows the feasibility of the routine collection of PRO data in patients with total hip and knee replacement. The use of PRO data provides opportunities for continuous quality improvement.

## Abbreviations

GEE: Generalized estimating equation analysis
HHS: Harris hip score
KSS: Knee society score
MCID: Minimal clinically important differences
OHS: Oxford hip score
PRO: Patient-reported outcomes
VAS: Visual analog scale
WOMAC: Western Ontario and McMaster Universities Arthritis Index
